# Primary cervicothoracic melanoma of spinal cord: a case report and literature review

**DOI:** 10.3389/fonc.2024.1417268

**Published:** 2024-05-28

**Authors:** Ying Dang, Aichao Du, Wenyuan Wei, Jungang Xue

**Affiliations:** ^1^ Department of Neurosurgery, Honghui Hospital, Xi’an Jiaotong University, Xi’an, Shaanxi, China; ^2^ Department of Neurosurgery, The Second Hospital of Lanzhou University, Lanzhou, China

**Keywords:** primary, cervicothoracic, spinal cord, electromyogram, CSEP, DSEP

## Abstract

A 53-year-old male patient presented progressive numbness and weakness in the right limbs for a 2-year duration. Magnetic resonance imaging scans revealed an intramedullary lesion crossed over cervical and thoracic levels accompanied by syringomyelia at the proximal end of the lesion. The patient underwent subtotal resection of the neoplasm. The histological findings of the tumor were consistent with primary intramedullary malignant melanoma and not initial ependymoma after careful dermatologic and ophthalmologic re-examination. Primary melanoma of the spinal cord, particularly cervicothoracic localization with syringomyelia, is seldom reported in the literature. We report a case of this uncommon tumor and also discuss the clinical course, diagnosis, and treatment.

## Introduction

Malignant melanomas are rarely seen as aggressive tumors that arise from the pigment-producing melanocytes. The World Health Organization classifies primary melanocytic tumors of the central nervous system (CNS) into meningeal melanomatosis, meningeal melanocytoma, meningeal melanoma, and meningeal melanocytosis (1). Primary melanomas of the CNS are rare. The occurrence of primary spinal melanoma is extremely rare, and only <70 cases have been reported in the literature since Hirschberg first reported primary spinal cord melanoma in 1906 (2). Spinal cord melanoma usually shows signal hyperintensity on T1-weighted images and signal hypointensity on T2-weighted images (3), with mild contrast enhancement of the lesion (4). However, the mass of our case appeared iso- and hypointense on T1-weighted images and non-homogeneous hypointensity on T2-weighted images accompanied by syringomyelia at the proximal end of the lesion. Moreover, the intramedullary melanoma in the presented case crossed over cervical and thoracic levels, which is seldom reported in the literature. Here, we present this uncommon case to add variety to clinical databases and discuss the clinical course, diagnosis, and treatment for primary spinal melanomas.

## Case description

### History and examination

A 53-year-old man was admitted to the hospital for progressive numbness and weakness in the right limbs for a 2-year duration. The neurological examination revealed 3/5 strength in the right arm and right leg with no other neurological positive signs. An electromyogram (EMG) showed a decreased rate and lengthened latent period of the F-wave on the median nerve and ulnar nerve in the right. Cortical somatosensory evoked potential (CSEP) and dermatomal somatosensory evoked potential (DSEP) revealed the disappearance of P1-wave, suggesting handicap of somatosensory conduction pathways of the right dorsal spinocerebellar tract under the C5 level. Beyond an elevated C-reactive protein level (179.48 mg/L, 0–5 mg/L), the results of routine blood chemical analysis and serum carcinoembryonic antigen were normal.

**Figure 1:**
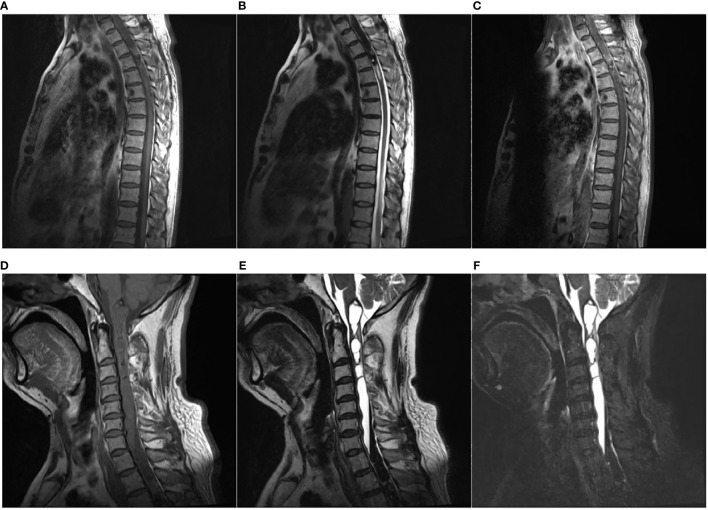
Magnetic resonance imaging (MRI). **(A–C)** An intramedullary tumor was located at C6–T4 with mixture of iso- and hypointensity on sagittal T1-weighted image (T1WI) **(A)**, non-homogeneous hypointensity on sagittal T2-weighted image (T2WI) **(B)**, and slightly non-homogeneous enhancement on sagittal T1WI with gadolinium **(C)**. **(D, E)** Long T1 [**(D)**, sagittal] and long T2 [**(E)**, sagittal] signals with increased signal intensity of fat-suppressed images [**(F)**, sagittal] at the level between medulla and C6 suggestive of syringomyelia at the proximal end of the lesion.

Magnetic resonance imaging (MRI) of the spine revealed a mixture of iso- and hypointense intramedullary mass at the C6–T4 level on T1-weighted images and non-homogeneous hypointensity on T2-weighted images (**Figures 1A, B**). After administration of contrast material, slightly non-homogeneous enhancement of the focal lesion at the T2–T3 level was observed (**Figure 1C**). Moreover, long T1 and long T2 signals with the increased signal intensity of fat-suppressed images at the level between the medulla and C6 indicated syringomyelia at the proximal end of the lesion (**Figures 1D–F**). The appearance of the lesion on MRI was misdiagnosed as ependymoma initially in this case.

### Operation and pathological examination

#### Operation

**Figure 2:**
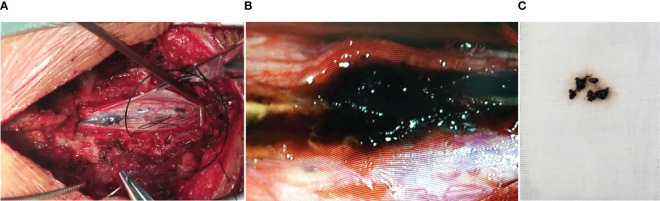
Intraoperative photograph showing the darkly pigmented intramedullary lesion with multiple small satellite lesions under the pia **(A)**. After spinal cord incision, the coal cinder-like lesions were removed in piecemeal, and multiple biopsies were taken **(B, C)**.

The patient underwent a C6–T4 laminectomy through a midline incision in the upper thoracic back. The epidural space was absolutely free of tumors with no pathological findings in the extravertebral soft tissues, spinous processes, or laminas. At the dural opening, a black tumor with multiple small satellite lesions under the pia was observed, and the main tumor was found to be apparently infiltrated with the parenchyma (**Figure 2A**). After the spinal cord incision, the coal cinder-like lesions were removed in piecemeal, and multiple biopsies were taken (**Figures 2B, C**). However, the lack of a clear cleavage plane between the tumor and normal tissue rendered surgical gross total resection (GTR) unachievable, and the tumor was debulked to the greatest possible extent.

#### Histopathological features

**Figure 3:**
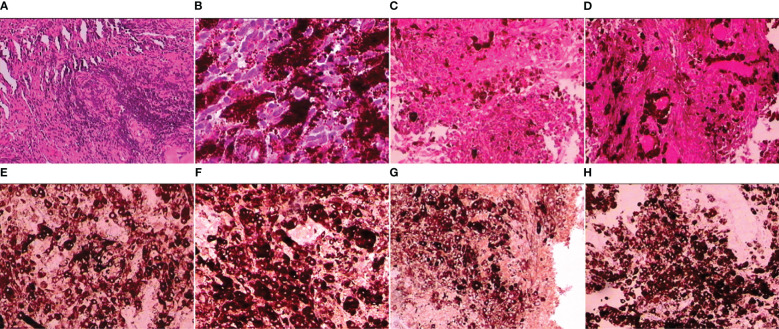
Neoplasm was densely cellular arranged in nests and sheets with deposition of abundant melanin pigment [**(A)**; hematoxylin and eosin (H&E), magnification ×100]; pleomorphic ovoid, spindle, or polygonal cells with significant nuclear atypia characterized by large nuclei, high nuclear-to-cytoplasmic ratio, and scattered mitosis [**(B)**; H&E, magnification ×200]; representative necrosis in the tumor [**(C)**; H&E, magnification ×200]; tumor cells with granular cytoplasmic pigmentation were arranged around the vascellum [**(D)**; H&E, magnification ×200]; positive staining for HMB-45, S-100, and MelanA [**(E–G)**; magnification ×200]; Ki-67 labeling indices counted more than 50% denoted pigmented cells that were mitotically active [**(H)**; magnification ×200].

Microscopic examination revealed that the tumor consisted of an abundance of ovoid, spindle, or polygonal tumor cells and was arranged in nests and sheets accompanying the deposition of abundant melanin granules (**Figure 3A**). Higher magnification of the lesions showed significant cellular pleomorphism with nuclear atypia and enlargement and a high nuclear-to-cytoplasmic ratio (**Figure 3B**). Necrotic areas were also seen, but no evidence of hemorrhage or products of its degeneration was identified, and tumor cells with granular cytoplasmic pigmentation were arranged around the vascellum (**Figures 3C, D**). Immunohistochemical examination showed that the neoplastic cells stained strongly positive for antimelanoma antibody (HMB-45), S-100, and MelanA, and Ki-67 staining showed high proliferative index (50%), both pointing to malignant melanoma of the spinal cord (**Figures 3E–H**).

#### Postoperative course

Subsequent examinations, including dermatological physical examination and ophthalmologic fundoscopic examination, endoscopy of the gastrointestinal tract, contrast-enhanced thoracoabdominal computed tomography (CT), and cranial MRI revealed no evidence of primary origin of the melanoma in other parts of the body. The final diagnosis of primary spinal malignant melanoma was confirmed. The patient refused any further aggressive or adjuvant treatment and was discharged to a rehabilitation facility. Postoperative telephone follow-up was performed periodically. At the last follow-up assessment, the patient had an uneventful recovery with no clinical deterioration and no additional complications after postoperative 6 months.

## Discussion

Although primary spinal melanoma is extremely rare, the high disability and mortality rate of this tumor have attracted more attention recently. Computer searches were performed on MEDLINE/PubMed (2018 to date), Embase (2018 to date), Cochrane Library, China National Knowledge Infrastructure (CNKI), Chinese Biomedical Database (CBM), and Wanfang databases. The search terms were as follows: spinal, Myelon, Cervical cord, Thoracic Cord, intramedullary, intra-medullary, and others. Here, recent literature on spinal cord melanoma was collected and systematically reviewed (**Table 1**), and a thorough review of the available literature was performed. There are some interesting points that need to be discussed, as follows.

**Table 1:** Literature review of primary malignant spinal cord melanoma.

Author (year)	Age	Sex	Location	Duration (months)	Symptoms	Treatment	EOR	Follow-up (months)	Outcome	Recurrence or metastases
Armocida D (5) (2018)	60	M	Thoracic	4	Radiating right side low back pain and paraesthesia	Surgery	GTR	1	Alive	No
Kohei Hironaka (6) (2019)	39	M	Lumbar and sacrum	—	Headache, papilledema, hydrocephalus, ventricular dilatation, aneurysm, progressive gait disturbance, dysuria	Surgery	Aneurysm clipping, ventriculoperitoneal (VP) shunt Codman	14	Died	No
Ritodhi (7) (2019)	78	M	Cervical and thoracic	2	Fatigue, increased right-side weakness, paresthesia, urinary incontinence	Surgery	GTR	18	Alive	No
Tang S (8) (2020)	55	M	Lumbar	36	Low back pain with toe paralysis and sensory disturbances	Surgery	GTR	5	Alive	Yes
Corrêa DG (9) (2020)	78	M	Thoracic	6	Progressive myasthenia was lower limb paralysis	Surgery+radiotherapy+chemotherapy	GTR	—	Alive	Yes
Akgun MY (10) (2020)	30	F	Cervical	Less than a month	Decreased sensation	Surgery	GTR	84	Alive	No
Hongbo Lv (11) (2021)	67	M	Thoracic	12	Progressive calf tremor and paroxysmal convulsions, numbness and weakness of the legs, paraplegia, and dysuria	Surgery	GTR	3	Alive	No
Our one	53	M	Cervical and thoracic	24	Progressive numbness and weakness of the limbs	Surgery	STR	12	Alive	No

M, male; F, female; EOR, extent of resection; GTR, gross total resection; STR, subtotal resection; —, unclear.

**1. Clinical features:** The clinical symptoms and signs of primary spinal cord melanoma are often non-specific. The presenting symptoms are predominantly those of spinal cord compression and neurological deficits, which are dependent on tumor location. Some patients may present with elevated intracranial pressure due to obstruction of the cerebrospinal fluid (CSF) circulation, although the occurrence of this is rare (12). The mean symptom duration in previous cases was 15 months and ranged from 0.3 to 96 months (13). Primary spinal malignant melanoma most frequently presents as a middle or lower thoracic cord lesion (14), probably because of the normally higher density of melanocytes in these locations. Primary spinal melanomas are usually located in the intradural extramedullary or intramedullary compartment. Less commonly, these tumors are extradural or spread out along the nerve root sheaths to involve the extradural tissue (15, 16). M. Zhang et al. reported that across 60 cases of primary spinal melanomas, 30.51% of the tumors were cervical, 52.54% were thoracic, and 16.95% were lumbar; 37.74% of the tumors were located intramedullary, and 62.26% were located extramedullary (2). Primary spinal melanoma can also give rise to metastases or diffuse leptomeningeal dissemination, although the occurrence of this is rare (17, 18). In this case, the 53-year-old male patient presented progressive numbness and weakness in the right limbs for a 2-year duration, and the intramedullary lesion was located at the C6–T4 level. A spinal cord lesion is not usually suspected at the presentation of the first symptoms; therefore, the diagnosis of a primary spinal melanoma may be delayed, and leptomeningeal dissemination may progress before a definitive diagnosis.

**2. Auxiliary examination:**

**2.1 MRI:** Primary spinal melanoma should be suspected when T1-weighted images show signal hyperintensity and when T2-weighted images show signal iso- or hypointensity, with mild contrast enhancement of the lesion (3). MRI may offer some indication of a melanotic lesion, but the rarity of a primary melanotic lesion in the CNS most often precludes the preoperative radiological diagnosis of this lesion. However, primary malignant melanoma varies in its imaging features, based on the degree of melanocytic content and the presence of hemorrhage and fat (19). In our case, the appearance of the lesion on MRI images was partly in accordance with the reported findings in the literature. T1-weighted images of our case revealed signal iso- and hypointensity at the C6–T4 level, and T2-weighted images showed signal non-homogeneous hypointensity (**Figures 1A, B**). After administration of contrast material, mild and non-homogeneous enhancement was observed in the foci of the lesion (**Figure 1C**). Different from previous cases, MRI images of our case appeared long T1 and T2 signals with increased signal intensity of fat-suppressed images at the level between the medulla and C6, which indicated syringomyelia at the proximal end of the lesion (**Figures 1D–F**). These differences in MRI signal intensities are related to the degree of the paramagnetic effects of stable free radicals in melanin and/or hemorrhagic products (20). The syringomyelia in this case was probably caused by obstructing cerebrospinal drainage by the spinal cord lesion. Nevertheless, the MRI pattern may correspond to that of other pigmented tumors, such as meningeal melanocytoma, melanotic schwannoma, or a tumoral hemorrhagic lesion (21). MRI patterns can easily suggest an erroneous diagnosis. In our patient, although the signal pattern on MRI was partly in accordance with that usually seen in occupying melanotic neoplasm, the long duration of symptoms, the rounded borders of the tumor, and the presence of an associated intramedullary cyst on MRI suggested an initial diagnosis of ependymoma. The ultimate diagnosis must be made following histopathologic examination.

**2.2 PET/CT:** In addition to MRI, positron emission tomography/CT (PET/CT) has become a useful imaging modality for the auxiliary examination of malignant melanomas (22). Indeed, some authors have reported the accuracy of PET as being almost 91% when diagnosing the local and distant involvement of malignant melanoma (23). A primary origin outside the spinal cord can be excluded after PET scanning (24), which plays a more and more important role in distinguishing the primary from the metastatic spinal melanoma.

**2.3 Gene examination:** Gene analysis on genomic DNA to determine the presence of possible oncogenic somatic mutations has become a new tool for early discovery and further targeted therapy of malignant melanoma. Some authors have reported that characteristic mutations in BRAF, NRAS, and CDKN2A are frequently seen in cutaneous melanoma (25). G. Angelino et al. reported a case of primary leptomeningeal melanoma with the presence of an NRAS^Q61K^ mutation (26).

**3. Histopathological features:** Histopathological analysis is indispensable for the confirmative diagnosis of primary spinal melanoma from other similar lesions. Malignant melanoma is characterized by positive immunohistochemical reaction to HMB-45, MelanA, and S-100 protein. Furthermore, the high Ki-67 labeling index indicates the high malignant potential of the lesions, which is a protein highly expressed in proliferating cells and encoded by the *MIB-1* gene. In addition, vimentin, Leu7, and epithelial membrane antigen (EMA) can be used to distinguish spinal melanoma from other different spinal tumors undergoing melanization, such as meningeal melanocytoma, meningioma, schwannoma, medulloblastoma, and gliomas (27). In our case, the immunohistochemical examination showed that the neoplastic cells stained strongly positive for HMB-45, S-100, and MelanA, and Ki-67 staining showed a high proliferative index (50%), pointing to a confirmative pathological diagnosis of malignant melanoma. Nevertheless, our immunohistochemical examination also showed positive EMA, cytokeratin pan (CK), and smooth muscle actin (SMA) staining, which suggested that the tumor may be heterologous (**Supplementary Figures 1A–C**). As far as we were able to determine, the strong positivity of characteristic markers such as HMB-45, S-100, and MelanA lent support to the pathological diagnosis of malignant melanoma because of the non-specificity of the markers EMA, CK, and SMA.

**4. Differential diagnosis:** According to Hayward’s criteria, diagnosis of a primary melanoma must consider the following factors: 1) malignant melanoma outside the CNS was not detected, 2) absence of this lesion in other sites in the CNS, and 3) the intramedullary lesion was confirmed pathologically (16). Our case was in accordance with these criteria. However, preoperative diagnosis of primary spinal melanoma is often difficult since the gross pathological, histological, and radiological features of the many varied spinal lesions overlap. The differential diagnosis of spinal pigmented lesions includes meningeal melanocytoma, metastatic malignant melanoma, and other uncommon melanotic tumors such as neurocutaneous melanosis, leptomeningeal melanomatosis, and melanotic schwannoma.

**4.1 Meningeal melanocytoma:** According to Brat, meningeal melanocytoma and malignant melanoma both arising from the normal melanocytic cells in the leptomeninges are the two extremes of a spectrum of primary melanocytic neoplasms ranging from low-grade to high-grade in the CNS (28). Melanoma and melanocytoma may be distinguished from each other on the basis of pathological features and clinical behavior (19). Pathologically, melanocytomas are well-differentiated tumors with benign histological features, and the lack of mitotic activity, nuclear pleomorphism, and hyperchromaticity is a characteristic that indicates melanocytoma rather than melanoma (29). Nevertheless, distinguishing between malignant melanoma and well-differentiated melanocytoma remains a diagnostic challenge. Hoffmann et al. recently suggested that molecular analysis is the best method for distinguishing between melanocytoma and malignant melanoma (30). Moreover, melanocytomas were usually cured by gross total resection alone with lower levels of local recurrence and mortality than melanomas (28).

**4.2 Metastatic malignant melanoma:** Although the CNS is a common site of metastases from malignant melanoma, which is the third most common neoplasm to metastasize to the CNS, spinal metastatic melanoma is extremely rare with accompanying multiple lesions in other sites of the CNS (31). K.D. Barron et al. reported malignant melanoma metastatic to the spinal cord and coverings that occurred only once in a series of 127 metastatic lesions of the spinal cord verified by autopsy (32). In addition, Z. Gokaslan et al. also reported intramedullary spinal cord metastasis that unusually occurred in as few as 2% of autopsy cases of systemic cancers and usually signified a late-stage event for the patient (33). Patients with melanoma metastatic to the CNS have a poor prognosis, with a median survival of 113 days after discovery (34). It is obviously important to determine if the melanoma is primary or secondary. However, in some cases, the primary tumor remains undetectable, and thus, it is difficult to differentiate metastases from primary spinal melanoma. A thorough physical examination to search for a primary cutaneous, mucosal, or ocular melanoma is recommended and is usually sufficient to exclude evidence of systemic disease (31). However, this is all the more difficult when one considers that achromic cutaneous melanomas exist, that metastatic melanomas of the skin can appear following the complete disappearance of a primitive melanoma, and finally that authentic primary melanomas of the central nervous system can metastasize elsewhere (35, 36). Moreover, Bergdahl et al. concluded that primary malignant melanomas of the CNS may also metastasize inside and outside the CNS (37). Recently, some authors have applied non-invasive PET/CT scanning to search for primary concealed malignant melanoma outside the CNS to substantiate the diagnosis (24).

**4.3 Other melanotic tumors**

**4.3.1 Neurocutaneous melanosis:** Neurocutaneous melanosis is one of the most infrequent neoplastic lesions of the CNS, which is characterized by the presence of congenital melanocytic cutaneous nevi associated with intracranial leptomeningeal melanocytosis. Compared with primary melanoma, neurocutaneous melanosis is associated with large or multiple congenital nevi, and the age of patients is usually younger (37).

**4.3.2 Leptomeningeal melanomatosis:** Primary leptomeningeal melanomatosis is a rare, diffuse neoplasm of the CNS that arises from melanocytes within the leptomeninges. It is also referred to as a meningeal variant of primary malignant melanoma (38). However, the morphological characteristics of leptomeningeal melanomatosis are diffuse darkening and thickening of the leptomeninges in the gross specimen. The CT and MR imaging also reveal diffuse thickening of the leptomeninges, with abnormal enhancement on the postcontrast images.

**4.3.3 Melanotic schwannoma:** Melanotic schwannomas are rare primary lesions in the CNS that consist of neoplastic Schwann cells and proliferating melanocytes (39). These lesions are more typically intracranial, but they also occur within the spinal canal. When they develop within the spine, the tumors most often arise in the thoracic region, and they may be intramedullary (40). Compared with spinal melanoma, melanotic schwannoma is usually well-circumscribed, and the behavior of this neoplasm is typically benign. Moreover, results from immunohistochemical staining help secure differential diagnosis. Melanotic schwannomas stain positive for S-100 protein Leu7 and vimentin but stain variably with glial fibrillary acid protein, HMB-45, and other melanocytic markers.

Other melanotic tumors like melanocytic glioma and medulloblastoma are very rare and are usually distinguished from spinal melanoma by pathological and immunohistochemical characteristics.

**5. Treatment:** Because of the rarity of primary spinal melanomas, the development of a standard treatment protocol is difficult. As case reports have accumulated, most clinicians have generally accepted the view that treatment for primary spinal melanoma needs multidisciplinary management (41).

Surgical GTR offers patients the greatest chance for survival (13). Unfortunately, most patients with intramedullary melanoma have many or diffuse lesions with ill-demarcated neural tissue, and GTR is not feasible. In our case, subtotal resection (STR) of the intramedullary tumor was achieved.

The role of adjuvant radiotherapy and chemotherapy for primary spinal melanoma is still controversial. Some authors have suggested radiotherapy and chemotherapy for preventing local tumor recurrence and dissemination combined with GTR or even STR (41), but others have claimed that the procedure may be ineffective and produce radiation-induced toxicity (19). To the best of our knowledge, adjuvant radiotherapy and chemotherapy should be integrated with surgical resection, especially subtotal resection, though malignant melanomas are considered to be highly radio-resistant tumors (42). Liang Wu et al. performed adjuvant radiotherapy in four STR patients, and three of them showed no recurrence during follow-up (24). Chemotherapy, including intrathecal administration, has been attempted. B.C. Bae et al. reported a favorable outcome of primary spinal melanoma with chemotherapy including vincristine, bleomycin, and cisplatin. Intrathecal methotrexate, interleukin-2, and dacarbazine (DTIC) have been reported for primary CNS melanomas and may be effective in controlling tumor progression for a certain period (43). However, there is little evidence that radiotherapy and chemotherapy are effective for primary spinal melanoma, and the efficacy remains to be assessed in a larger series.

Immunotherapies such as interferon-α, interferon-γ, and lymphokine-activated killer (LAK) cells have been applied to other melanomas (43). Additionally, polyvalent melanoma vaccine has also been tried in malignant melanoma patients with minimal residual disease after resection of the tumor, which shows encouraging results with prolonged survival of several years (44). Newly emerged targeted immunotherapy has shown some positive effects. Ganesh et al. reported a series of metastatic spinal melanoma patients who underwent regular immunotherapy and acquired longer median survival (45). Zhang et al. reported that the use of PD-1 or PD-L1 antibodies has some effect in preventing tumor local recurrence of spinal malignant melanomas (46). This new management can be attempted in primary CNS melanomas.

**6. Prognosis**: In general, the prognosis of primary spinal melanoma varies depending on the site of the initial primary and the presence or absence of other visceral involvement (31). The survival duration of patients with primary spinal melanoma ranged from 3 to 156 months (12, 14). The mean survival time of patients who have undergone surgical excision of spinal cord melanoma with or without additional treatment is 6 years 7 months after the onset of symptoms (41). According to a retrospective study, the 12-month survival was 89.6% and the 72-month survival was 39.6% (2). These tumors are potentially malignant and liable to recurrences locally, or at a distance within the CNS (47), and they rarely metastasize to outside or inside the central nervous system. Leptomeningeal seeding and hydrocephalus are poor prognostic factors in melanomas, which means disease progression and difficulty in archiving total removal of the melanoma (48). Beyhan et al. suggested that the prognosis for dural melanoma is better than that for leptomeningeal melanoma because of no involvement of the leptomeninges (49).

**7. Conclusion:** In conclusion, although primary spinal melanomas are rare, we should suspect this tumor if the MRI depicts a spinal cord tumor with paramagnetic properties. Surgical removal with multidisciplinary management is recommended. To the best of our knowledge, the presented report is unique in that the intramedullary lesion crossed between cervical and thoracic levels and showed iso- and hypointense on T1-weighted images accompanying syringomyelia at the proximal end of the lesion. The final diagnosis was primary spinal melanoma based on the results of pathological examination. Further studies with larger sample sizes are required to collect more imaging data and establish well-defined diagnostic criteria and treatment strategies.

## Data availability statement

The datasets presented in this study can be found in online repositories. The names of the repository/repositories and accession number(s) can be found in the article/**Supplementary Material**.

## Ethics statement

The studies involving humans were approved by Ethics Committee of Xi’an Honghui Hospital. The studies were conducted in accordance with the local legislation and institutional requirements. The participants provided their written informed consent to participate in this study. Written informed consent was obtained from the individual(s) for the publication of any potentially identifiable images or data included in this article.

## Author contributions

YD: Investigation, Writing – original draft, Writing – review & editing. AD: Writing – review & editing. WW: Supervision, Writing – review & editing. JX: Supervision, Writing – review & editing.

## Supplementary material

Supplementary Figure 1 Positive staining for epithelial membrane antigen (EMA), cytokeratin pan (CK), smooth muscle actin (SMA) [**(A–C)**, magnification: 200×].
