# Supplementary figures and images for: Primary cervicothoracic melanoma of spinal cord: a case report and literature review
Source: Front Oncol. 2024 May 28;14:1417268. doi: 10.3389/fonc.2024.1417268 (PMC11165122; doi:10.3389/fonc.2024.1417268)

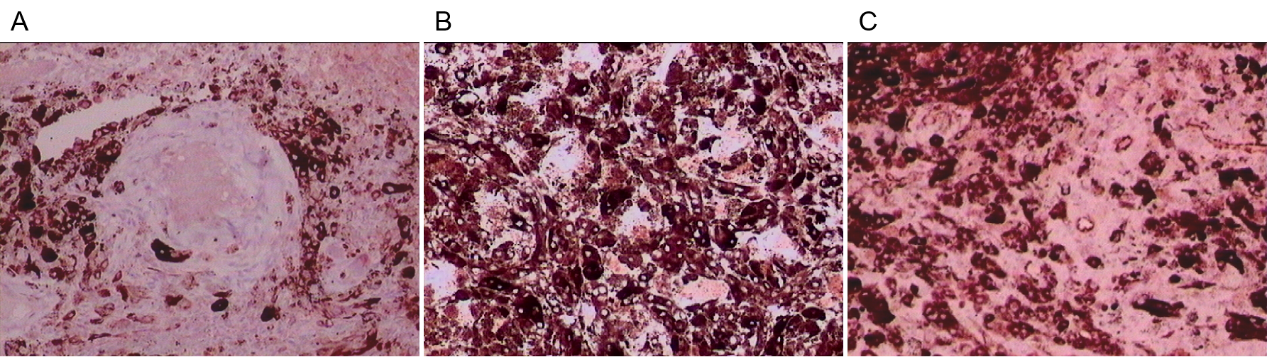

Supplement: Supplementary Figure 1 — Positive staining for epithelial membrane antigen (EMA), cytokeratin pan (CK), smooth muscle actin (SMA) [(A–C), magnification: 200×]. [file Image_1.tif]
